# Pathologist workload, work distribution and significant absences or departures at a regional hospital laboratory

**DOI:** 10.1371/journal.pone.0265905

**Published:** 2022-03-25

**Authors:** Michael Bonert, Uzma Zafar, Raymond Maung, Ihab El-Shinnawy, Asghar Naqvi, Christian Finley, Jean-Claude Cutz, Pierre Major, Anil Kapoor

**Affiliations:** 1 Division of Anatomical Pathology, Department of Pathology and Molecular Medicine, Staff Pathologist—St. Joseph’s Healthcare Hamilton, McMaster University, Hamilton, Ontario, Canada; 2 Rutgers Health/St Barnabas Medical Center, Livingston, New Jersey, United States of America; 3 Department of Pathology and Laboratory Medicine, Staff Pathologist—Royal Inland Hospital, University of British Columbia, Kamloops, British Columbia, Canada; 4 Division of Thoracic Surgery, Department of Surgery, Staff Thoracic Surgeon—St. Joseph’s Healthcare Hamilton, McMaster University, Hamilton, Ontario, Canada; 5 Division of Medical Oncology, Department of Medicine, Staff Medical Oncologist—Hamilton Health Sciences, McMaster University, Hamilton, Ontario, Canada; 6 Division of Urology, Department of Surgery, Staff Urologist—St. Joseph’s Healthcare Hamilton, McMaster University, Hamilton, Ontario, Canada; University of Waterloo, CANADA

## Abstract

**Objective:**

Assess the work environment of salaried pathologists via (1) the national workload system (L4E), (2) work distribution among/in three hospital groups, and (3) the frequency of significant absences or departures (SADs).

**Methods:**

Automated analysis of pathology reports from a regional laboratory (accessioned 2011–2019) using validated computer code.

**Results:**

The study set contained 574,099 pathology reports, reported by 63 pathologists. The average yearly L4E workload units/full-time equivalent for three hospital groups were 8,101.6, 6,906.5 and 4,215.8. The average Gini coefficient for full-time pathologists in the three hospital groups were respectively 0.05, 0.16 and 0.23. The average yearly SADs rates were respectively 13%, 16% and 9%. The group with the highest SADs rate had the intermediate Gini coefficient and intermediate workload.

**Conclusions:**

High individual workload and work maldistribution appear to be associated with SADs. Individual workload maximums and greater transparency may be essential for limiting staff turnover, maintaining high morale, and efficient laboratory function with a high quality of care.

## Introduction

Health policy shapes the care that patients get and the healthcare work environment. In regard to the latter, it is generally known that employee turnover is reflective of the work environment [1]; however, it is not known how workload and workload distribution affects turnover in pathology.

In environments where healthcare providers are salaried or on contract with no link to workload, such as anatomical pathology, work distribution may not be well tracked, as it has little to no bearing on compensation and, thus, unlikely to be audited/independently scrutinized for accuracy. The indirect result may be a significant maldistribution of work–that may arise due to multiple factors, which may include the lack of information, exploitation of knowledge asymmetry and/or power relationships.

### Measuring workload and work distribution

To gauge (professional) workload, workload models have been developed. In Canada, the *Level 4 Equivalent (L4E)* workload system was developed and endorsed by the Canadian Association of Pathologists [2].

In the United Kingdom, The Royal College of Pathologists workload model [3] acknowledges the issue of distribution; it states that the model is intended to “*facilitate equitable distribution of work among pathologists within a department*”

Remarkable is that a “clarification” [4] was issued later that practically undermined the idea of equity and/or the point of measuring work. The “clarification” stated: “*[The model] was not intended to provide a tool whereby pathologists may limit the amount of work they do on a daily basis*, *especially not in a way that may potentially harm patients*. *In its third edition the guidance moved from retrospective calculation of ‘workload points’ to prospective calculation*. *This was intended to facilitate the equitable prospective distribution of workload between pathologists*, *not to justify stopping work after a specific number of points had been delivered on any one day*.”

### Impact of maldistribution and excess work

Work maldistribution is a significant issue. At the societal level and at the hospital level, an equitable workload and efficiency are highly desirable. If pathologists within an institution are inequitably overworked and under-worked: (1) quality will suffer from individuals that are overworked, as excessive work is known to adversely affect quality in pathology [5–7], which of course can lead to patient harm, (2) burnout and turnover rate among the overworked individuals is likely higher; these have costs to society as well as the individual pathologists and their families, (3) the underworked deliver poor value and may have insufficient work volume to maintain skills, and (4) the knowledge of significant workload inequality may have a negative impact on morale, especially among the individuals/groups with the higher workload.

Workload has been a flash-point in Canadian pathology for a long time [8–10], as pathologists are traditionally salaried. This means that a mechanism to address increased workload has to exist–or it leads to an inevitable stress on pathologists when work increases–be that due to increased volume, increased complexity or both. In the United States, laboratory administrations (due to economic incentives) demanded workloads that were not safe; this ultimately led to legislation (CLIA 1998) [11].

### Study objective

In this study, we sought to examine (professional) pathology workload and workload distribution. We believe they are both important for patient safety, optimal resource allocation and work environment desirability (or lack thereof), as may be inferred by significant absences and departures.

### Assessing inequality

Inequality can be quantified with a number of measures. The Gini coefficient is one well established measure [12]. It has been used widely to assess income distribution and wealth distribution around the world. It has also been applied to resource distribution in healthcare [13].

The Gini coefficient is a number that varies from zero to one. Zero represents complete equality (equal income or equal wealth or equal resources), and one represents maximal inequality (one person or entity receives all income or all resources or possesses all wealth).

Inequality can also be quantified by the amount of resources that would need to be redistributed to achieve equality.

Hoover [14] did this; he calculated the percentage of wealth that would have to be redistributed in an unequal population to achieve an equal one. This is known as the “Hoover index”. As wealth redistribution was done by the legendary outlaw “Robin Hood”, the “Hoover index” is also known as the “Robin Hood index”.

## Methods

Ethics approval (Hamilton integrated Research Ethics Board, Project ID: 4879) was obtained to assess workload using all pathology reports accessioned between January 1, 2011 and December 31, 2019 at a regional laboratory. Consent to analyze the data was not applicable as the data was fully anonymized.

All pathology reports were extracted in a standardized text format from the laboratory information system (MEDITECH). After the data was extracted, custom computer code, written in python (www.python.org), did the following: (1) captured the patient name and removed it, and (2) scrubbed all the patient identifiers, to generate anonymized pathology reports.

The anonymized reports were read by a second program to generate L4E workload units. The program also extracted the W2Q workload system numbers [15], the number of (tissue) blocks, the number of cases, and the total “shadow billings” (as per the Ontario Schedule of Benefits–March 2020) [16].

At this juncture it should be noted that: the Schedule of Benefits “shadow billings” are a tabulation completely unrelated to the pathologist’s compensation. Pathologists in the regional lab are all salaried and earn the same uniform level of compensation; the compensation is completely uninfluenced by the actual work done–that is considered within this analysis.

The output of the second program was read by a third program which replaced the healthcare providers with a unique anonymous identifier, coded the cases in a form that is readable by a program written in R (cran.r-project.org), and generated a completely anonymized data file for auditing purposes.

Details regarding the L4E (2018) workload analysis are further elucidated in a prior work. It was found that L4E system most accurately reflects the workload in pathology and thus used in this study [15].

### Definition of the pathologist groups

The regional laboratory is an amalgamation of the laboratory services for two regional hospital organizations (Hamilton Health Sciences and St. Joesph’s Healthcare Hamilton). Anatomical pathologists are present at four hospital sites (Hamilton General Hospital, McMaster University Medical Centre, Juravinski Hospital, St. Joseph’s Healthcare Hamilton).

The pathologist groups were defined based *only* on the origin of the cases the group members finalized in a given year. To simply the analysis and maintain greater anonymity: two smaller groups were lumped together.

The formal definition of the pathologist group (calculated for each year) was as follows:

1. if the pathologist’s fraction of surgical cases in the year for hospital organization alpha was greater than the pathologist’s fraction of surgical cases in the year for hospital organization beta: assign pathologist to group alpha; else: assign to group beta
2. if a pathologist was allocated to group beta: they were further subclassified into beta1 and beta2; if the pathologist’s fraction of cases for beta1 was greater than beta2: assign pathologist to beta1; else: assign to beta2

Pathologists remained anonymous throughout the analysis. “Moves” of pathologists between the groups were solely determined by the yearly case mix (alpha, beta1, beta2). Pathologists in group alpha, beta1, and beta2 were mapped into the numbered groups (1, 2, 3); to maintain anonymity the mapping is not given.

The workload of a pathologist group for a given year was the sum of the work of members in that group that year. The individual workload of a pathologist in a year was the work of all cases they placed the finalizing signature on; it was not based on who the case was assigned to or where the case originated from.

### Statistical analyses

R (r-cran.org) was used to further process the data and generate plots. The Gini coefficient was calculated using the developing code in the ’DescTools’ library [17] (https://github.com/cran/DescTools/blob/master/R/StatsAndCIs.r). The Gini function was run on test cases and recalculated a public data set. Summary statistics were generated with the ‘psych’ package [18] (https://cran.r-project.org/web/packages/psych/).

#### FTE adjustment for equal work

The relative FTE adjustment for equal work was calculated for the three groups, such that all groups have the same amount of work. Details of the calculation are found in S1 Appendix.

Positive FTE adjustments imply a deficit of FTEs in the group, i.e. more FTEs are required. Negative FTE adjustments imply a surplus of FTEs in the group, i.e. less FTEs are required.

#### Full-time equivalent (FTE)

In the context of this study, full-time equivalent (FTE) pathologist was defined as follows:

1. One FTE, if the pathologist signed cases >41 International Standards Organization (ISO) weeks per calendar year
2. ISO weeks signing/48 weeks FTEs, if the pathologist signed <42 weeks in the calendar year; example: a pathologist signed cases in 24 weeks of the year–they would be: 0.5 FTE (24/48 = 0.5)

The FTE definition was developed to capture all staff that sign cases. It does not rely on employment records that may or may not be complete. The 48-week cut-off was chosen as contract pathologists typically have 4 weeks off. The 42-week cut-off was chosen as full-time employees have six weeks or more time off.

#### Significant absences or departures (SAD)

Significant absence or departure rate (SADR) was defined as follows:

*SADR = Σ (SAD events) / FTEs*

Where:

- FTEs = full-time equivalents (as defined above)
- SAD event = a pathologist signing more than 13 ISO weeks less than the prior year AND (NOT having a SAD event in the previous year)

The term ‘SAD’ was coined as it cannot be determined from the data whether (1) an individual pathologist has taken a significant leave from signing cases (i.e. is absent) *or* (2) has departed from the institution. SAD events in consecutive years were excluded to avoid the double counting of departures that happen part way through a calendar year.

## Results

After a preliminary analysis was done, cancer review cases (~700-800/year) and cases referred in from external laboratories (~3500/year) were excluded, as information found in the other cases was lacking. All other surgical pathology and cytopathology cases were included; this yielded 574,099 reports. Workload data could be extracted from 574,093 reports.

The custom code could reliably classify cases. Approximately 1,100 randomly selected cases were audited by five different pathologists to assess the accuracy of the automated analysis. The accuracy for the L4E workload scoring was ~95%.

### Workload measures and pathologist groups

**Fig 1:**
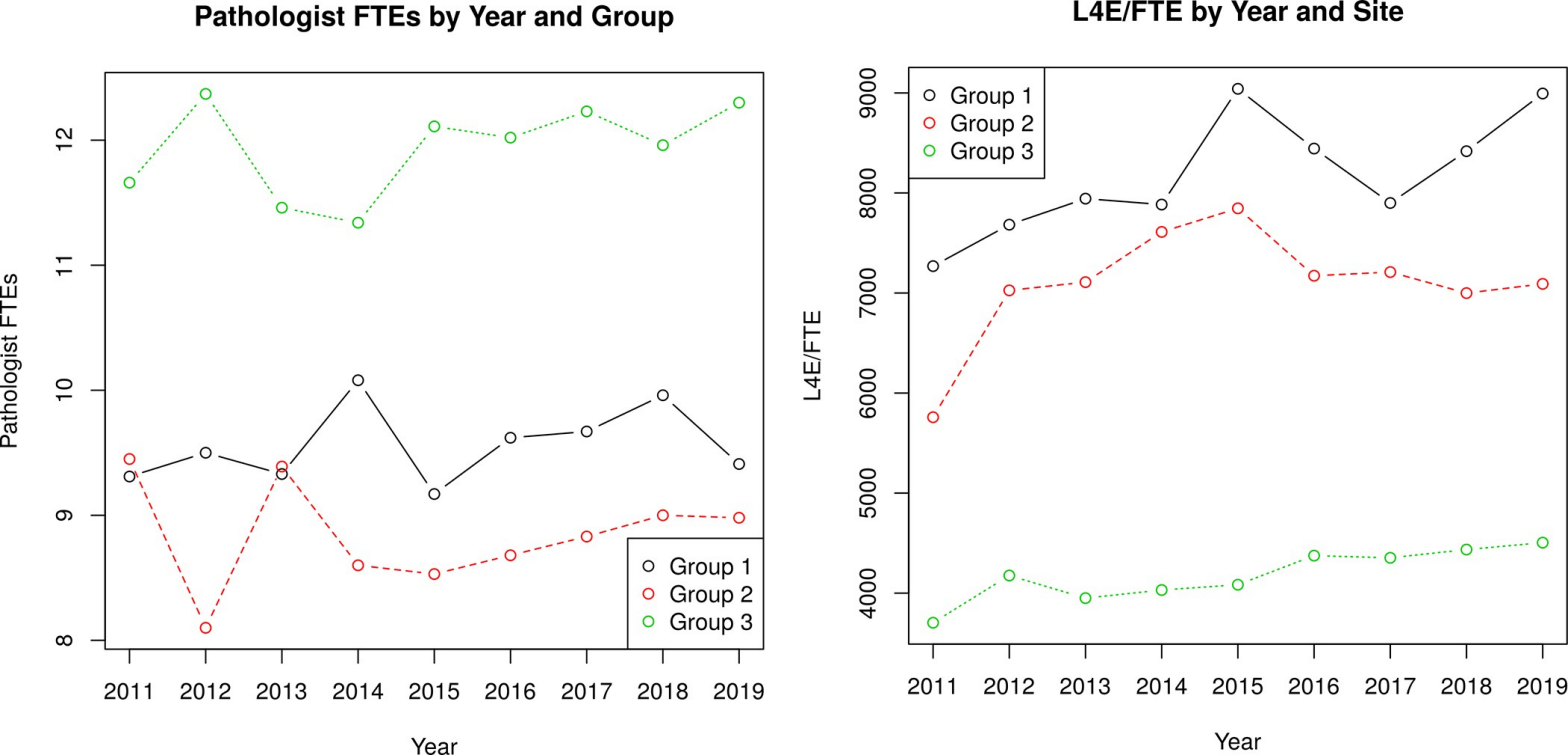
a: Number of full-time equivalents (FTEs) by group and year. b: Level 4 equivalent (L4E) workload units per full-time equivalent FTE by group and year.

The number of full-time equivalent (FTE) pathologists in all hospital sites together was relatively stable over the study period, as decreases in some sites were often seen in conjunction with increases in others (see Fig 1A).

Workload increased per FTE, as measured by L4E workload units (see Fig 1B/S1a Table in S1 File).

There were also significant differences between the pathologist groups. The least amount of work was in group 3 (3704.0 L4E workload units per FTE) and the greatest amount of work was in group 1 (9041.2 L4E workload units per FTE). The L4E/FTE difference was 4489.7/year or 100% higher in relation to group 3; the L4E/FTE was 8994.3/year for group 1 and 4504.6/year for group 3 in 2019 –further details in the S1a Table in S1 File.

### Variation within pathologist groups

To assess workload variation within the groups, the pro rata yearly L4E workload units/FTE were calculated and then used to generate summary statistics (see Table 1, S1b.1-S1b.3 Table in S1 File).

**Table 1:** Assessing variation within the groups—summary statistics—L4E workload units.

Group	n	mean	sd	median	min	max
Group 1	10.9	8101.6	1015.0	8135.2	5936.8	9466.0
Group 2	10.9	6906.5	1974.5	7241.5	3155.2	9840.5
Group 3	14.3	4215.8	1673.2	4232.4	1206.8	6528.9

Numbers shown are the average of the yearly parameters (2011–2019). The year-by-year numbers are within S1b.1-S1b.3 Table in S1 File. The workload was calculated *pro rata* yearly; for example, if a pathologist worked only half of a year their workload for that time period would be divided by 0.5 to arrive at the workload on a yearly basis.

‘n’ = number of pathologists signing cases, ‘mean’ = L4E units for given year, ‘sd’ = standard deviation of L4E for the group, ‘median’ = median L4E for group of pathologists, ‘min’ = minimum L4E in the group, ‘max’ = maximal L4E in the group.

Pathologists groups showed marked differences in the parameters (L4E workload units, Ontario Schedule of Benefits (shadow billing) fees) collected. Significant workload variation was seen within pathologist groups, as seen with the summary statistics.

Different than the group-based comparison, the pro rata yearly (Ontario) Schedule of Benefits (shadow billing) fees/FTE varied much more significantly. The maximum and minimum (Ontario) Schedule of Benefits (shadow billing) fees/FTE (for pathologists >0.3 FTE) differed by greater than 11x. The individual with the highest (Ontario) Schedule of Benefits (shadow billing) fees did a high volume of small specimens; these are compensated at approximately twice the rate per L4E workload unit (details are provided in S1c.1-S1c.3 Table in S1 File).

To further assess the inequality of work distribution, the Gini coefficient was calculated for yearly L4E workload units/FTE for the full-time pathologists (see Table 2). The Gini coefficient for all pathologists >0.3 FTE pro rata is provided in the S2b Table in S1 File.

**Table 2:** Gini coefficient by year, full-time only (L4E workload).

Group 1 FT (L4E Gini)	Group 2 FT (L4E Gini)	Group 3 FT (L4E Gini)	All Groups FT (L4E Gini)
0.049	0.156	0.227	0.203

‘FT’ = Full-time, defined in manuscript text; Gini Coefficient for members in the group—measures inequality of L4E within group. The year-by-year data is with S2a Table in S1 File.

#### FTE adjustment for equal work

The number of FTEs that would have to be redistributed to have equal work distribution among the groups was calculated; it is shown in Table 3 and more details are provided in the S2e Table in S1 File.

**Table 3:** Group inequity—Robin Hood (RH) Full-Time Equivalent (FTE) pathologists.

RH FTEs Group 1	RH FTEs Group 2	RH FTEs Group 3
3	1	-4

The number of full-time equivalent (FTE) pathologists that are required to balance the workload between the three groups (1, 2 and 3). Positive numbers indicate additional pathologists are required. Negative number indicate a relative surplus of pathologists. The numbers above are the averages of the numbers year-by-year; the year-by-year numbers are within a S2e Table in S1 File.

The FTE adjustment for equal work shows that marked differences exist between the groups. Group 1 and Group 2 have a relative deficit of FTEs, as indicated by positive values. Group 3 has a relative surplus of FTEs, as indicated by a negative value.

The average number of redistributed FTEs required for equal work was +3, +1 and -4 for group 1 to 3 respectively. If these numbers are expressed as percentages of the workforce in the individual groups, the increases required are +30%, +13% and -33% for the group 1 to 3 respectively.

### Significant absences or departures

In the nine-year period, a total of 63 pathologists signed cases. During the study period, the number of full-time equivalents (FTEs) fluctuated between 30.0 and 31.0. The number of SADs was 30 for 2012 to 2019 –detailed breakdown provided in S3a Table in S1 File. Thus, the SAD rate for the 8-year period (2012–2019) was ~100%—see Table 4.

**Table 4:** Significant absences or departures rate by year and group.

SADs Rate Group 1	SADs Rate Group 2	SADs Rate Group 3
0.131	0.161	0.094

The significant absences or departures (SADs) rate is the number of SADs by the number of pathologists in the group by FTEs. The formal FTE definition is within the manuscript. The numbers above are the averages of yearly rates for each group. The numbers year-by-year are within S3b Table in S1 File.

## Discussion

The random audits done suggest that the analysis herein is sufficiently accurate to predict significant trends.

The marked variation of (Ontario) Schedule of Benefits (shadow billing) fees suggests a large cleavage in the broader pathology community. Small specimens are very heavily favoured by the (Ontario) Schedule of Benefits. This likely leads to very significant salary differentials on the basis of equal work (as measured by L4E workload units) when comparing (1) fee-for-service pathologists doing small specimens to (2) pathologists (predominantly working in hospitals) on a fixed salary.

### Significant absences or departures

The “significant absence or departure rate” was developed to indirectly gauge staff turnover, based on the information within the laboratory information system.

The “significant absence or departure” rate definition captures individuals that: decrease their workload yet do not vacate their position (e.g. a person that is on long-term disability, extended stress leave, parental leave). It does not require tracking employment relationships. It also more heavily weights absences or departures that are not replaced quickly, as the (FTE as calculated above) denominator decreases. Thirteen ISO weeks was chosen as the cut-point, as it represents a quarter year.

#### Staff turnover (departure rate)

Novis *et al*. [19] studied staff turnover (departures per staff and per time) using a survey and found the median 3 year turnover was 14% in 14 US institutions. The 90^th^ percentile three-year turnover rate was 28%.

If the SADR is adjusted to a 3 year period (as used by Novis *et al*.), it is ~37.5% (~100% / 8 years x 3 years = 37.5%). SADR is not the same as turnover; however, it appears to be the closest metric that has been published on. The SADR appears to be high, in comparison to the published turnover rate by Novis *et al*.

The highest SADR was seen in group 2. It has a relatively high Gini coefficient (in relation to group 1) and a relatively high workload (in relation to group 3). The next highest SADR is seen in group 1, the group with the highest workload.

High or very high workload with or without inequality appears to drive SADs. High inequality in the presence of low workload appears to have a lesser effect.

#### FTE adjustment for equal work (“Robin Hood FTEs”)

To achieve equal workload: (1) work and/or (2) FTEs would have to be shifted between the groups.

In the wealth distribution context, Hoover [14] described the fraction that would need to be shifted between the upper half and lower half of the income distribution. This subsequently became known as the “Robin Hood index”.

The number of FTEs was chosen over the fraction of work, as it is likely more intuitive. These FTEs could be called “Robin Hood FTEs” (as they are analogous to the “Robin Hood index”); they represent a redistribution from relatively under-worked pathologist groups to relatively over-worked pathologist groups.

The sum of the “Robin Hood FTEs” across the laboratory as a whole is zero, as it represents a redistribution of work force resources.

### Limitations

Biomarkers (e.g. estrogen receptor status in breast cancer, epidermal growth factor receptor (EGFR) status in lung adenocarcinoma, mismatch repair in colorectal cancer) are frequently reported by pathologists that do not finalize the case. These individuals may do significantly more work; however, this is not captured in the current analysis. Separating the workload for cases done by several pathologists would add considerable complexity to the analysis; thus, this represents a limitation.

The workload points of formal consults within the regional laboratory (that involve reviewing the whole case and filling out the “Consultation” section) are assigned to the primary pathologist. Thus, individuals that do a larger volume of consults (in relation to other pathologists) are disadvantaged. The effect of this simplification is likely small; however, it may be significant for a small subset of pathologists.

The analysis does not capture academic contributions in relation to the clinical workload. A prior analysis of academic productivity may be informative [20]. A way to combine the findings herein with those findings and other academic authorships would generate a more complete picture of work in the environment.

The analysis does not capture outside pathology work and consultations. It is known that several pathologists “moonlight” (work a second job for additional pay) at other hospitals in the region. This is part of the public record (as hospital privileges are public information via the College of Physicians and Surgeons of Ontario); however, it was not considered within this analysis. Consultations are captured in the Ontario Health Insurance Plan (OHIP) billings and accessible via access to information requests [21]; however, merging that data with the data herein is not possible due to the way the ethics protocol was written. In the context of patient safety, the extra (paid) voluntary work may not be relevant, as the providers (by the nature of the work being voluntary) can self-regulate. Also, the psychological impact of *extra [nonvoluntary] work that is not compensated* is, arguably, quite different than *additional work which is paid and voluntary*.

Provider anonymization precludes an examination of how the workload and workload distribution may be associated with characteristics that are surrogates of power/influence (e.g. gender, age, seniority, ethnic background, educational background) and the leadership structure.

### Summary of findings

Major findings are that: the environment has (1) significant workload maldistribution, and (2) marked staff turnover (a large number of departures and large number of new hires). Further, the SAD rate and the SADs distribution in the study period suggests on-going challenges with retention, and a practice environment characterized by frequent disruptions.

### Possible underlying causes

It is acknowledged that the lack of workload information may be an explanation for the workload distribution seen. As insiders working within the system, this seems to be unlikely; the paucity of objective published data appears to be no accident. We suspect the findings herein have their nidus in policy and organizational structures.

#### System organization and policy

The unequal distribution of work may be due to organizational structures in hospitals that allows individuals in the system to shape the system to their advantage. Prior work by Dossa *et al*. showed pay inequality among fee-for-service surgeons along gender lines [22]. This could be explained by power differentials and would be in keeping with the presence of systemic bullying—that can be considered endemic in Canadian medicine and in the words of a *Canadian Medical Association Journal* article “starts at the top” [23, 24]. It would also be compatible with the lack of effective representation, and the illegality of formal representation; it is illegal for pathologists (and other physicians) to unionize in the local environment (Ontario) [25, 26].

In 2017, the regional government (The Ontario Government) initiated review for labour law reform recommended physicians and surgeons that are employees should be able to unionize [25, 26].

Pathologists are excluded from large parts of the Employment Standards Act (in Ontario); a pathologist could legally be asked to work every minute of the week without breaks and would not be entitled to any overtime pay [27].

The policy makers’ justification for this appears to be the perception that professionals universally have decision latitude in their employment relationship; this is far from the reality for rank and file pathologists. Pathologists generally have no control over the volume of their work; no workload maximums exist and there is no established mechanism to hand-off excessive work. Pathologists frequently deal with time sensitive diagnoses where a delayed diagnosis/care can have a very negative impact on patient outcome.

To some degree, the sense of professionalism and sense of duty to report cases in a timely manner for patient care by pathologists, avoids delays in reports; however, this is at the expense of the pathologists’ personal time and patient safety—by working unpaid overtime. As work hours are typically not tracked, the (chronic) overtime may go unrecognized until a crisis is reached. A recent survey showed that Canadian pathologists on average work 48.9 hours per week, suggesting there are 8.9 hours of uncompensated overtime with the accompanying medicolegal risks [28]. Perhaps unsurprisingly, multiple crises in Canadian pathology have arisen, and a number of these have resulted in public inquires that have linked systemic/organizational issues and workload to patient suffering and preventable deaths [29–31].

It has previously been shown that human resources in pathology have not increased with the workload in the past decades [15, 32]. Likely, this is as anatomical pathology is mostly funded through global hospital budgets; it is not directly and transparently tied to the workload and service provision (unlike in the fee-for-service context). Hospital pathologists generally do not bill for *in hospital work*. Unlike fee-for-service providers, additional work can be foisted on hospital pathologists without any additional compensation–a situation unique to salaried and contract physicians. From a fiscal perspective, hospitals have strong incentives to pressure their salaried/contract employees to deliver, may demand too much (as workload safe guards are lacking) and thereby potentially precipitate a crisis or adverse events. In the context of overwork and adverse events, despite moral culpability, the hospital’s legal risk may be limited, as pathologists are generally medico-legally responsible for the cases they sign-out. In the current funding paradigm, workload maldistribution may sustain workforce under-funding that may endanger patients (in a vicious cycle with high turnover and poor performance), as the division leadership may be isolated from the overwork.

### A local manifestation of a global challenge?

Globally, pathologists are asked by health system stakeholders and payers to justify the funding allocation to deliver a standard of service. While health systems around the world differ dramatically in their organization and funding, the fundamental task of measuring work and assessing value is similar as many tasks in pathology do not differ. Granular real-world data sets that include parameters frequently used in workload models (e.g. case type, origin of tissue, number of blocks, ancillary tests) are important in this dialogue. This study analyzes a large data set from a regional laboratory and makes it available for further analyses.

One of the United Nations’ Sustainable Development Goals is “ensuring healthy lives and promoting well-being for all” [33]; in this context, the distribution of *health resources* is important if greater health *for all* is to be achieved. How can this “for all” ideal be achieved? We think part of the answer is: greater *transparency*.

The World Health Organization (in its 2015 Accountability Framework [34]) defines *transparency* as “an organization’s openness about its activities, providing reliable and timely information that is accessible and understandable on what it is doing, where and how its activities take place, and how the organization is performing, unless the information is deemed confidential.” We believe greater transparency in pathology workload (and the allocation of health resources more generally) is needed. The inequality of resource distribution presented herein appears to benefit the few over the many; if the type of information presented herein is accessible to all health system stakeholders in each locale it would generate pressure for a more equitable resource distribution that likely better serves more healthcare providers and better serves the public at large.

## Conclusions

Laboratory information system data allows insight into the practice environment of pathologists. High workload per FTE and unequal work distribution appears to be associated with staff turnover. Workload at the level of the individual pathologist should be assessed with transparency. High workload for the individual pathologist, may be a marker for poor quality in a practice. Workload maldistribution likely represents a suboptimal resource allocation and may be a marker of powerful individuals in the system making the system work for themselves.

Health policy that assures similar pay for similar work would likely (1) address conditions that decrease morale and divide healthcare providers, and (2) reduce healthcare provider burnout and optimize resource use while improving patient outcomes.

## Supporting information

S1 Fig (PDF)

S1 File (PDF)

S1 Appendix (DOCX)

S1 Data (7Z)
