# Supplementary material for: Pathologist workload, work distribution and significant absences or departures at a regional hospital laboratory
Source: PLoS One. 2022 Mar 25;17(3):e0265905. doi: 10.1371/journal.pone.0265905 (PMC8956155; doi:10.1371/journal.pone.0265905)
Supplement: S1 Fig — (PDF) [file pone.0265905.s001.pdf]

Supplemental Figures

Pathologist Workload, Work Distribution and Significant Absences or Departures at a Regional Hospital Laboratory

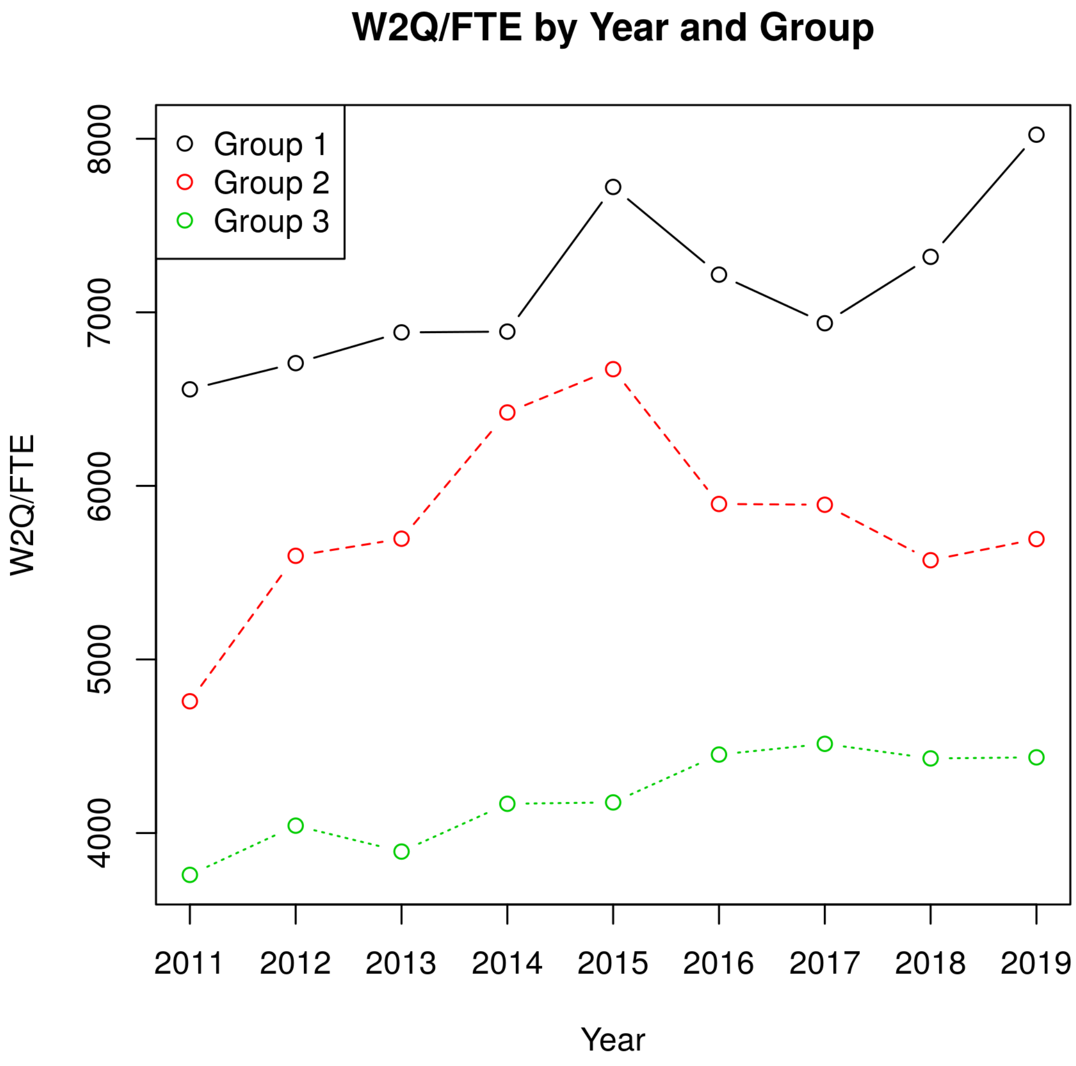

Figure S1a: Work2Quality (W2Q) units per FTE by group and year.

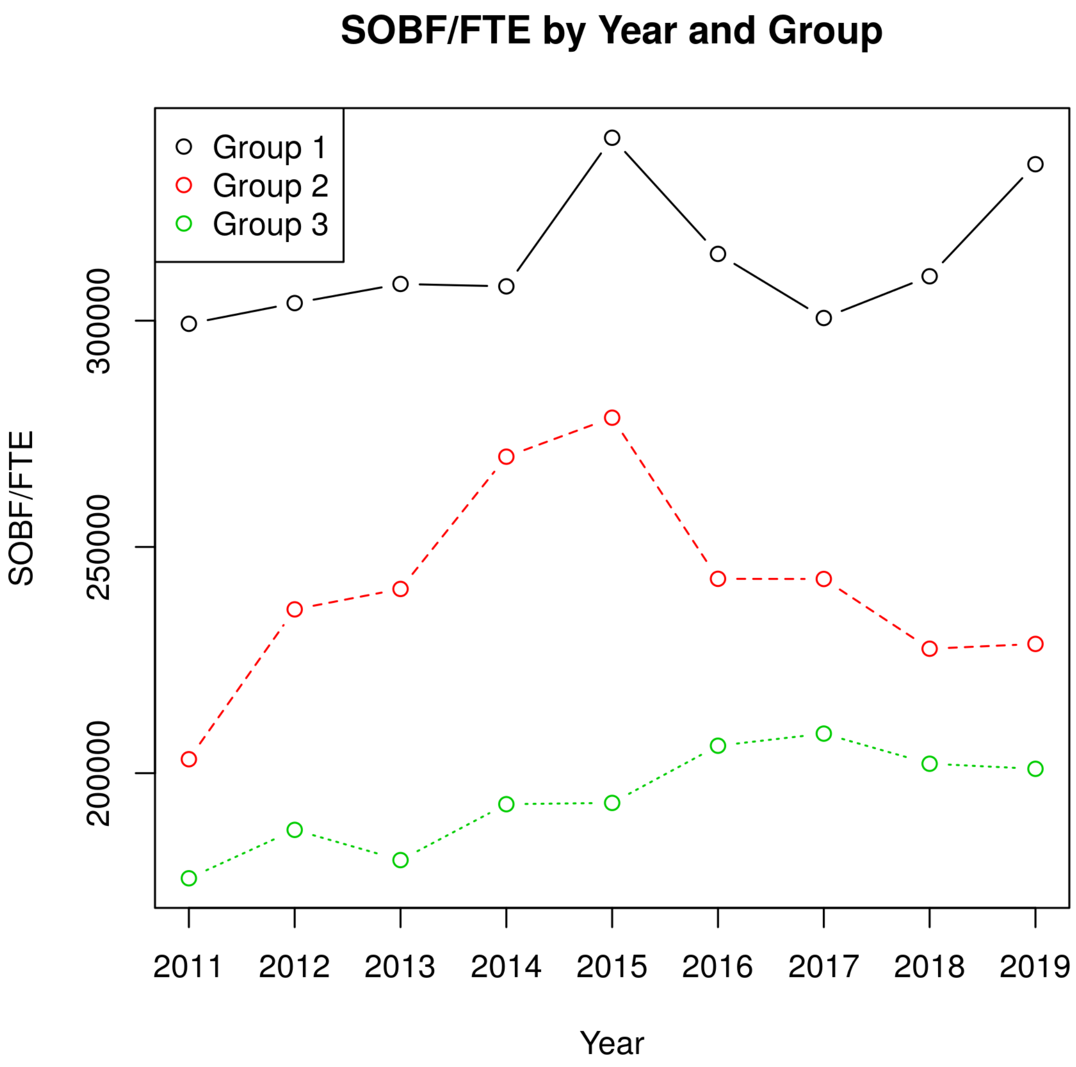

Figure S1b: Schedule of Benefits Fees (SOBF) in dollars per FTE by group and year.

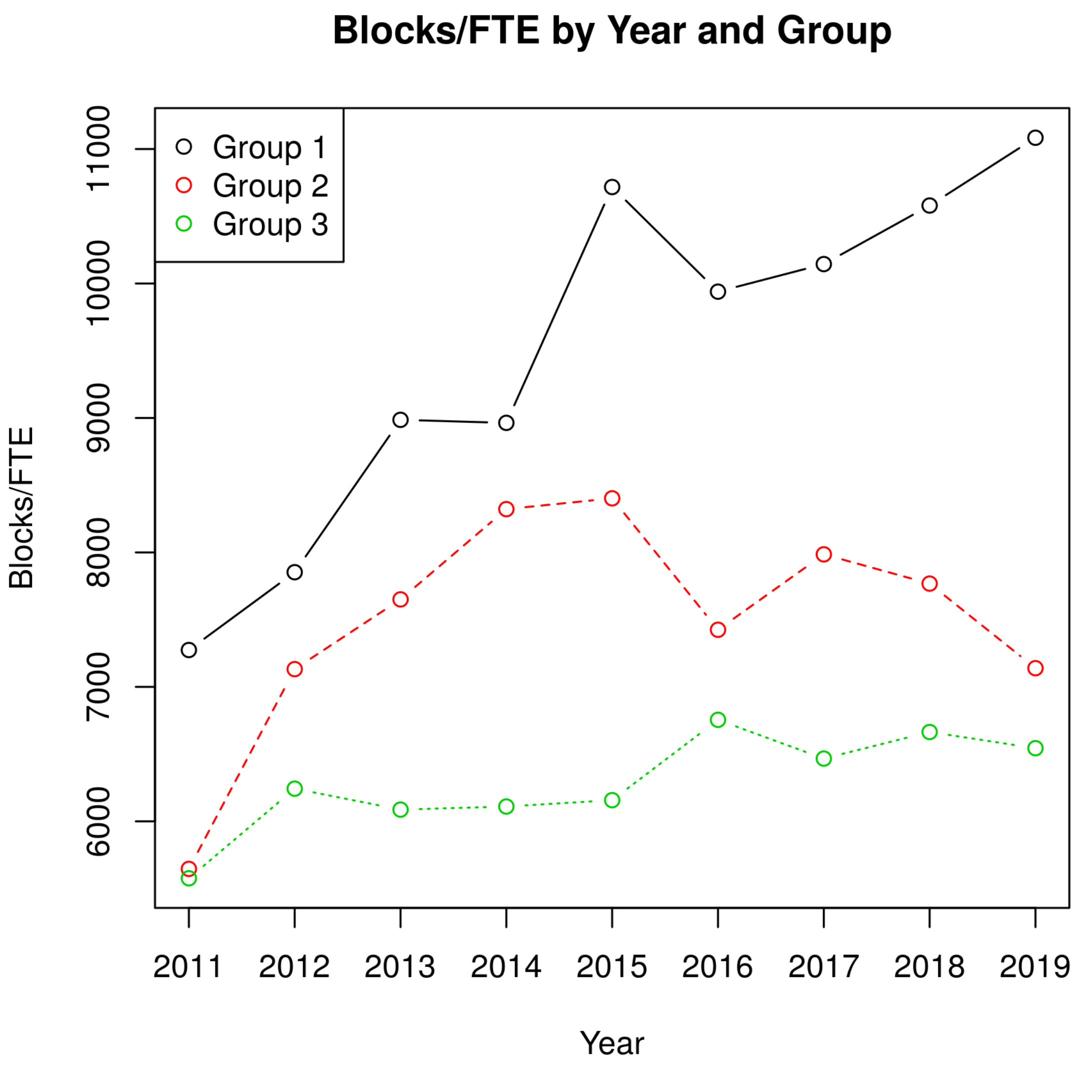

Figure S1c: Blocks per FTE by group and year.

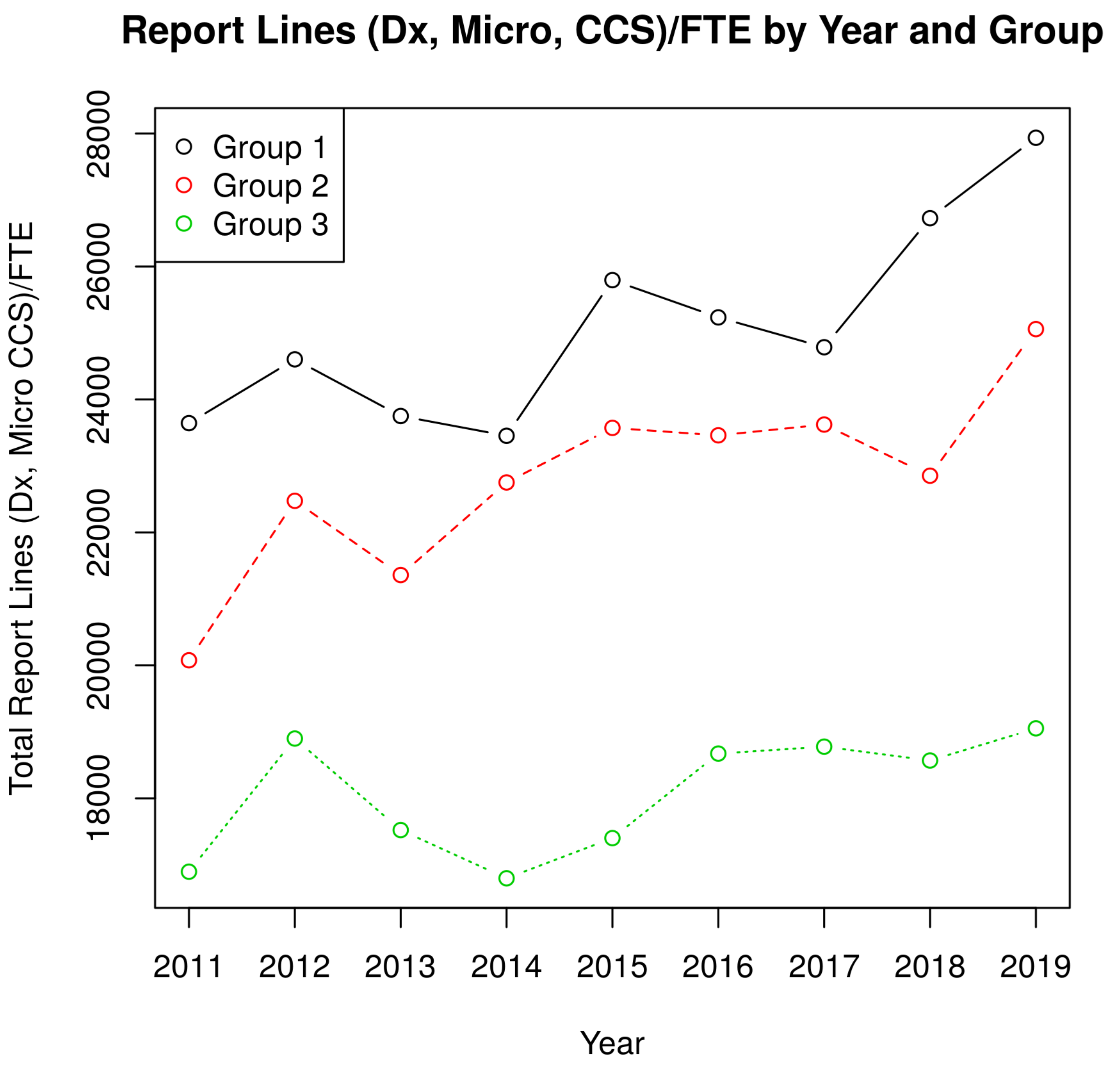

Figure S1d: Number of report lines (in diagnosis section, micro section and CCS section) per FTE by group and year.

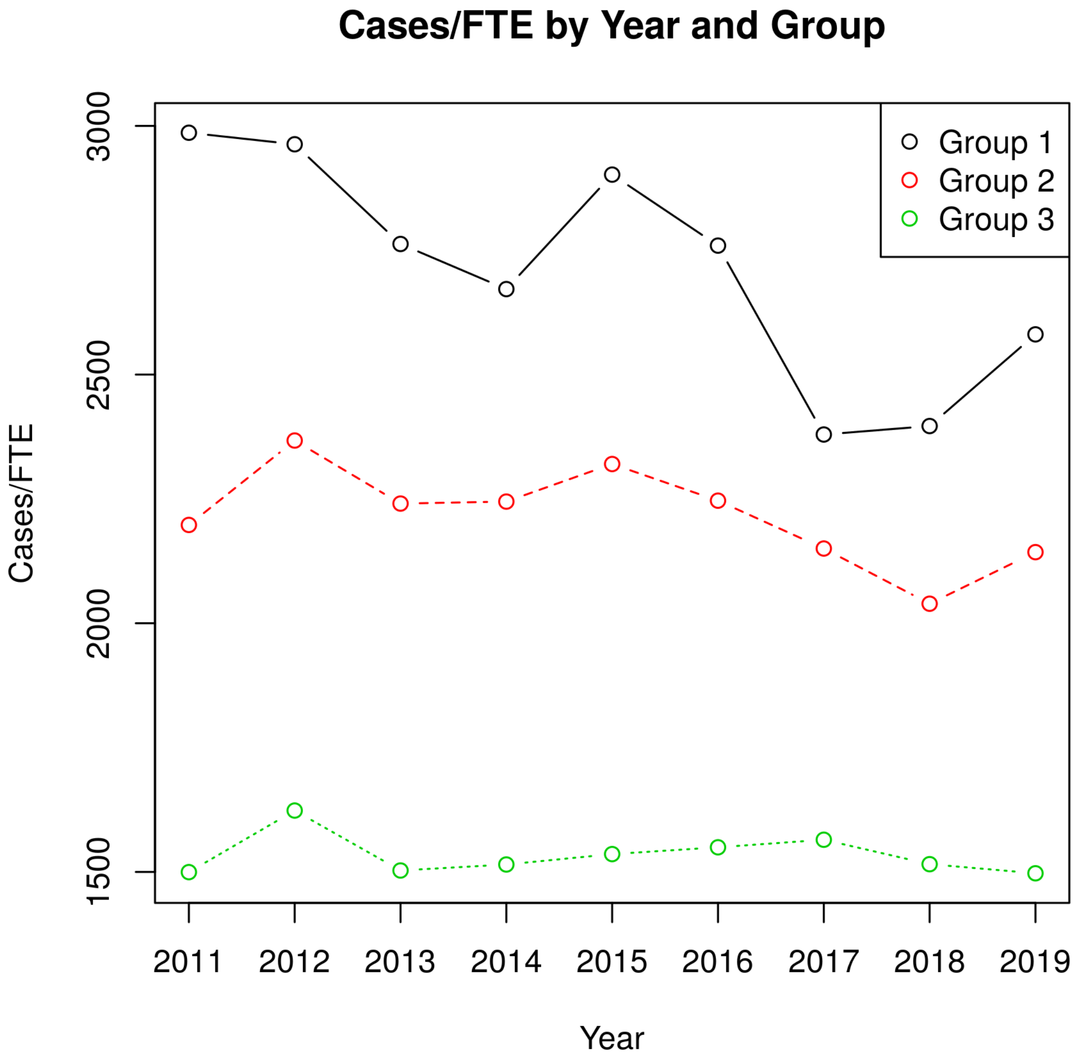

Figure S1e: Number of cases per FTE by group and year.
