# Supplementary material for: Pathologist workload, work distribution and significant absences or departures at a regional hospital laboratory
Source: PLoS One. 2022 Mar 25;17(3):e0265905. doi: 10.1371/journal.pone.0265905 (PMC8956155; doi:10.1371/journal.pone.0265905)
Supplement: S1 File — (PDF) [file pone.0265905.s002.pdf]

Supplemental Tables

Pathologist Workload, Work Distribution and Significant Absences or Departures at a Regional Hospital Laboratory

Table S1a: L4E Workload Units per Full-Time Equivalent by Group

Year	Group 1 L4E/FTE	Group 2 L4E/FTE	Group 3 L4E/FTE
2011	7268.8	5758.2	3704.0
2012	7683.1	7026.3	4176.0
2013	7943.5	7108.6	3950.0
2014	7884.4	7610.6	4031.0
2015	9041.2	7846.3	4084.0
2016	8444.0	7172.8	4374.9
2017	7899.6	7209.4	4352.9
2018	8417.6	6999.1	4435.5
2019	8994.3	7091.1	4504.6
Average	8175.2	7091.4	4179.2

This table shows the workload per full-time equivalent on the basis of the pathologists within each group.

The data within this table is plotted in *Figure 1b*.

Numbers in in *Table S1a* are close to the 'mean' values in *Table S1b.1*, *Table S1b.2* and *Table S1b.3*; they differ as the calculation is different.

The numbers in *Table S1a*/*Figure 1b* are calculated from the *total work in a group* divided by *the number of full-time equivalents*.

The numbers in *Table S1b.1*, *Table S1b.2* and *Table S1b.3* are calculated from the *pro rata yearly L4Es per pathologist in each group*.

Table S1b.1 - Summary Statistics - L4E Workload System - Group 1

Year	n	mean	sd	median	trimmed	mad	min	max	range	skew	kurtosis	se
L4E 2011	10	7121.2	1181.5	7291.4	7272.9	919.1	4340.0	8688.9	4348.9	-0.99	0.46	373.6
L4E 2012	11	7503.9	1212.2	7668.7	7750.2	653.3	4131.8	8659.8	4527.9	-1.82	2.56	365.5
L4E 2013	11	7939.7	702.3	7886.8	7987.2	813.5	6545.3	8906.6	2361.3	-0.41	-0.97	211.7
L4E 2014	11	7841.8	958.0	7779.2	7838.4	1106.1	6107.2	9607.4	3500.2	0.03	-0.74	288.8
L4E 2015	12	9036.3	965.8	9285.0	9219.1	298.4	6253.8	9990.5	3736.7	-1.84	2.91	278.8
L4E 2016	11	8234.8	939.7	8273.1	8352.5	632.2	6067.8	9342.3	3274.5	-0.92	0.01	283.3
L4E 2017	10	7926.3	624.2	7724.1	7932.2	463.7	6998.8	8806.3	1807.4	0.25	-1.53	197.4
L4E 2018	11	8383.1	890.6	8190.6	8352.5	1392.5	7240.0	9801.9	2561.9	0.12	-1.59	268.5
L4E 2019	11	8927.3	1661.0	9118.1	9007.2	1010.3	5746.1	11390.1	5644.1	-0.23	-0.89	500.8
Average	10.9	8101.6	1015.0	8135.2	8190.2	809.9	5936.8	9466.0	3529.2	-0.65	0.02	307.6

Table S1b.2 - Summary Statistics - L4E Workload System - Group 2

Year	n	mean	sd	median	trimmed	mad	min	max	range	skew	kurtosis	se
L4E 2011	11	5713.2	1089.6	5939.9	5822.3	931.9	3227.5	7216.9	3989.4	-0.76	-0.02	328.5
L4E 2012	11	6737.1	2028.0	7041.3	6868.9	1761.1	3167.4	9120.6	5953.2	-0.66	-0.97	611.5
L4E 2013	13	6500.8	2136.5	6452.5	6526.8	2366.2	2848.1	9867.7	7019.7	-0.17	-1.14	592.6
L4E 2014	11	7082.5	2161.3	7236.3	7108.1	2147.0	3533.1	10401.7	6868.5	-0.34	-1.18	651.7
L4E 2015	13	7809.4	2640.2	8908.3	7917.6	2354.9	3229.2	11199.2	7970.0	-0.55	-1.21	732.3
L4E 2016	11	7447.3	2012.7	7832.0	7474.1	1330.1	3228.5	11424.6	8196.1	-0.15	0.19	606.9
L4E 2017	9	7207.7	1927.4	7313.6	7207.7	475.0	3176.4	10265.3	7088.9	-0.53	-0.10	642.5
L4E 2018	9	6999.1	1793.7	7251.0	6999.1	1091.9	3306.0	10005.1	6699.0	-0.44	-0.09	597.9
L4E 2019	10	6661.0	1981.4	7198.5	6858.3	1205.4	2680.3	9063.0	6382.8	-0.88	-0.62	626.6
Average	10.9	6906.5	1974.5	7241.5	6975.9	1518.2	3155.2	9840.5	6685.3	-0.50	-0.57	598.9

Table S1b.3 - Summary Statistics - L4E Workload System - Group 3

Year	n	mean	sd	median	trimmed	mad	min	max	range	skew	kurtosis	se
L4E 2011	15	3979.7	1651.1	3908.4	4057.2	2037.3	787.2	6165.5	5378.3	-0.25	-1.27	426.3
L4E 2012	14	4283.7	1491.1	4331.0	4324.3	1799.2	1569.7	6510.5	4940.8	-0.13	-1.43	398.5
L4E 2013	14	3862.0	1464.8	3866.3	3932.6	1680.4	1223.2	5653.6	4430.4	-0.50	-1.07	391.5
L4E 2014	15	4313.8	1495.1	4903.4	4359.3	1647.0	1495.7	6540.6	5045.0	-0.22	-1.32	386.0
L4E 2015	15	4106.8	1651.5	3891.5	4140.9	1989.2	1352.4	6419.1	5066.7	-0.10	-1.52	426.4
L4E 2016	13	4369.9	1798.8	3765.7	4415.6	2157.4	1413.5	6824.4	5410.8	-0.04	-1.63	498.9
L4E 2017	14	4231.5	1707.8	3980.1	4282.7	1829.1	1334.3	6513.3	5179.0	0.02	-1.58	456.4
L4E 2018	13	4421.9	1851.8	4799.6	4471.7	2302.2	1096.1	7200.1	6103.9	-0.17	-1.44	513.6
L4E 2019	16	4372.7	1946.6	4645.7	4460.1	2031.2	589.4	6933.2	6343.9	-0.54	-0.86	486.6
Average	14.3	4215.8	1673.2	4232.4	4271.6	1941.4	1206.8	6528.9	5322.1	-0.21	-1.35	442.7

The summary statistics were generated with the ‘psych’ package (https://cran.r-project.org/web/packages/psych/).

The numbers in the “Average” row of *Table S1b.1*, *Table S1b.2*, and *Table S1b.3* are found in *Table 1a* of the manuscript.

The workload was calculated *pro rata* yearly; for example, if a pathologist worked only half of a year their workload for that time period would divided by 0.5 to arrive at the workload on a yearly basis.

Table S1c.1 - Summary Statistics - Ontario Schedule of Benefits Fees - Group 1

Year	vars	n	mean	sd	median	trimmed	mad	min	max	range	skew	kurtosis	se
2011	1	10	$292,348	$53,399	$297,494	$300,450	$28,771	$160,818	$359,057	$198,239	-1.23	0.97	$16,886
2012	2	11	$297,640	$50,217	$311,411	$308,094	$35,384	$159,903	$341,291	$181,388	-1.73	2.26	$15,141
2013	3	11	$311,227	$29,138	$316,373	$311,660	$10,272	$254,346	$364,215	$109,869	-0.38	-0.28	$8,785
2014	4	11	$305,204	$35,983	$313,073	$303,861	$35,666	$253,486	$369,013	$115,527	0.10	-1.27	$10,849
2015	5	12	$341,453	$34,782	$345,381	$347,349	$19,156	$242,360	$381,592	$139,232	-1.75	2.68	$10,041
2016	6	11	$308,286	$36,372	$311,160	$309,100	$27,638	$235,434	$373,805	$138,371	-0.20	-0.46	$10,967
2017	7	10	$301,209	$19,889	$302,004	$301,320	$27,588	$272,360	$329,172	$56,812	0.00	-1.63	$6,289
2018	8	11	$309,795	$26,906	$314,876	$309,160	$30,720	$273,923	$351,376	$77,452	-0.11	-1.58	$8,113
2019	9	11	$347,015	$117,534	$337,477	$326,060	$57,125	$212,576	$670,043	$457,467	1.70	2.37	$35,438

Table S1c.2 - Summary Statistics - Ontario Schedule of Benefits Fees - Group 2

Year	vars	n	mean	sd	median	trimmed	mad	min	max	range	skew	kurtosis	se
2011	1	11	$199,873	$45,982	$206,518	$205,659	$37,102	$89,979	$257,690	$167,711	-1.02	0.31	$13,864
2012	2	11	$226,516	$74,970	$241,594	$229,319	$64,341	$97,874	$329,932	$232,058	-0.36	-1.02	$22,604
2013	3	13	$229,846	$74,787	$249,823	$229,885	$78,282	$112,545	$346,714	$234,170	-0.18	-1.36	$20,742
2014	4	11	$256,541	$82,961	$271,890	$257,631	$61,627	$110,963	$392,303	$281,340	-0.32	-0.86	$25,014
2015	5	13	$272,321	$101,892	$308,152	$280,092	$77,100	$65,591	$393,568	$327,977	-0.63	-0.92	$28,260
2016	6	11	$256,319	$77,518	$259,394	$250,544	$35,109	$119,858	$444,756	$324,898	0.78	1.07	$23,372
2017	7	9	$242,997	$55,549	$247,198	$242,997	$17,245	$110,441	$312,375	$201,934	-1.24	0.91	$18,516
2018	8	9	$227,501	$55,356	$221,064	$227,501	$21,364	$112,167	$310,939	$198,772	-0.53	-0.24	$18,452
2019	9	10	$213,651	$66,303	$238,632	$222,157	$16,979	$74,763	$284,483	$209,721	-1.06	-0.44	$20,967

Table S1c.3 - Summary Statistics - Ontario Schedule of Benefits Fees - Group 3

Year	vars	n	mean	sd	median	trimmed	mad	min	max	range	skew	kurtosis	se
2011	1	15	$203,208	$105,406	$182,228	$195,018	$92,548	$57,780	$455,105	$397,325	0.83	-0.14	$27,216
2012	2	14	$194,064	$73,600	$191,059	$191,589	$95,649	$96,006	$321,823	$225,816	0.16	-1.63	$19,670
2013	3	14	$179,004	$75,245	$156,458	$177,805	$111,299	$61,357	$311,047	$249,689	0.10	-1.36	$20,110
2014	4	15	$211,704	$80,883	$251,029	$207,014	$154,831	$98,518	$385,865	$287,347	0.38	-0.97	$20,884
2015	5	15	$199,395	$80,464	$174,165	$198,995	$81,880	$70,649	$333,339	$262,690	0.24	-1.31	$20,776
2016	6	13	$207,704	$80,560	$158,894	$207,117	$87,118	$100,134	$321,736	$221,603	0.12	-1.92	$22,343
2017	7	14	$203,734	$80,672	$164,138	$203,994	$82,892	$97,240	$307,107	$209,867	0.19	-1.89	$21,561
2018	8	13	$204,233	$75,300	$185,709	$200,567	$93,924	$106,726	$342,071	$235,345	0.27	-1.47	$20,884
2019	9	16	$198,199	$75,136	$191,342	$201,742	$87,767	$60,077	$286,724	$226,648	-0.40	-1.17	$18,784

The summary statistics were generated with the ‘psych’ package (https://cran.r-project.org/web/packages/psych/).

Pathologists in the regional lab are salaried or on contract and earn the same base uniform level of compensation (based on their FTE status).

The dollar amounts show above are “shadow billings”; these billings are unrelated to the actual payment for the services rendered.** The amounts are calculated from the specimens that the pathologists signed-out; they show the work maldistribution. Internal consultations were not captured in the tallied Ontario Schedule of Benefits fees.

The SOB fees analysis was done in 2020 dollars. The SOB changed over the course of the study period. This was not taken into account, nor was the change in the cost of living. A more detailed analysis would considered these effects. We suspect these effects are modest and do not significantly affect the trends or possible conclusions.

** “Shadow billing” in this context is closest to the definition used by the Government of Saskatchewan (Shadow Billing – Mode 0 & 9 Practitioners, Billing information sheet, Revised September 2020, Saskatchewan, Available at: https://www.ehealthsask.ca/services/resources/establish-operate-practice/Documents/Billing%20Information%20Sheet%20-%20Shadow%20Billing.pdf): “Shadow billing is a term used for practitioners who are not billing fee-for service, and whose submissions to Medical Services Branch [the provincial health insurer] do not generate an actual ‘payment’.”

Table S2a: Gini Coefficient by Year, Full-Time Only (L4E Workload)

Year	Group 1 FT (L4E Gini)	Group 2 FT (L4E Gini)	Group 3 FT (L4E Gini)	All Groups FT (L4E Gini)
2011	0.057	0.132	0.269	0.196
2012	0.033	0.143	0.233	0.202
2013	0.052	0.166	0.220	0.206
2014	0.058	0.166	0.230	0.200
2015	0.019	0.272	0.219	0.256
2016	0.031	0.155	0.251	0.199
2017	0.044	0.161	0.236	0.184
2018	0.069	0.140	0.193	0.196
2019	0.074	0.065	0.191	0.184
Average	0.049	0.156	0.227	0.203

Table S2b - Gini Coefficient for Pro Rata Yearly L4E Workload System

Year	Group 1 (L4E Gini)	Group 2 (L4E Gini)	Group 3 (L4E Gini)	All Groups (L4E Gini)
2011	0.090	0.107	0.244	0.199
2012	0.077	0.172	0.202	0.202
2013	0.052	0.193	0.220	0.224
2014	0.070	0.178	0.202	0.208
2015	0.050	0.194	0.237	0.240
2016	0.064	0.149	0.242	0.204
2017	0.046	0.147	0.235	0.208
2018	0.063	0.140	0.244	0.204
2019	0.109	0.163	0.257	0.241
Average	0.069	0.160	0.231	0.214

Table S2c - Gini Coefficient for Pro Rata Yearly Ontario Schedule of Benefits Fees

Year	Group 1 (SOBF Gini)	Group 2 (SOBF Gini)	Group 3 (SOBF Gini)	All Groups (SOBF Gini)
2011	0.096	0.127	0.292	0.218
2012	0.082	0.191	0.220	0.193
2013	0.050	0.192	0.246	0.204
2014	0.070	0.187	0.213	0.178
2015	0.049	0.216	0.235	0.206
2016	0.068	0.155	0.220	0.173
2017	0.040	0.115	0.225	0.164
2018	0.051	0.136	0.216	0.171
2019	0.163	0.162	0.217	0.227
Average	0.074	0.165	0.232	0.193

Table S2d - Gini Coefficient by Year, Full-Time Only (Ontario Schedule of Benefits Fees)

Year	Group 1 FT (SOBF Gini)	Group 2 FT (SOBF Gini)	Group 3 FT (SOBF Gini)	All Groups FT (SOBF Gini)
2011	0.054	0.145	0.260	0.202
2012	0.043	0.150	0.209	0.184
2013	0.045	0.168	0.221	0.178
2014	0.058	0.190	0.206	0.168
2015	0.017	0.267	0.189	0.204
2016	0.036	0.137	0.233	0.166
2017	0.040	0.128	0.220	0.146
2018	0.056	0.136	0.184	0.164
2019	0.065	0.052	0.170	0.156
Average	0.046	0.153	0.210	0.174

‘year’ = calendar year cases were accessioned, ‘FT’ = full-time pathologist (1 FTE defined in manuscript text), Gini Coefficient for members in the group - measures inequality of SOBF within group

Table S2e: Robin Hood Full-Time Equivalent (FTE) Pathologists

Year	RH FTEs Group 1	RH FTEs Group 2	RH FTEs Group 3
2011	3.1	0.6	-3.7
2012	2.5	1.3	-3.8
2013	2.7	1.4	-4.1
2014	2.4	1.7	-4.1
2015	3.2	1.5	-4.7
2016	2.9	0.9	-3.9
2017	2.5	1.3	-3.8
2018	3.0	0.7	-3.8
2019	3.3	0.6	-4.0
Average	2.9	1.2	-4.0

The number of full-time equivalents (FTE) pathologists that are required to balance the workload between the three groups (1, 2 and 3). Positive numbers indicate additional pathologists are required. Negative number indicate a relative surplus of pathologists. The numbers within the manuscript are rounded to the nearest whole number.

Table S3a – Number of Significant Absences or Departures by Year and Group

Year	Group 1	Group 2	Group 3
2012	1	4	2
2013	1	2	1
2014	1	0	2
2015	2	1	1
2016	1	4	0
2017	1	0	1
2018	1	0	1
2019	2	0	1
Sum	10	11	9

Table S3b: Significant Absences or Departures Rate by Year and Group

Year	Group 1	Group 2	Group 3
2012	0.105	0.494	0.162
2013	0.107	0.213	0.087
2014	0.099	0.000	0.176
2015	0.218	0.117	0.083
2016	0.104	0.461	0.000
2017	0.103	0.000	0.082
2018	0.100	0.000	0.084
2019	0.213	0.000	0.081
Average	0.131	0.161	0.094

The significant absences or departures (SADs) rate is the number of SADs by the number of pathologists in the group by FTEs. The formal FTE definition is within the manuscript. The number of FTEs is calculated on a yearly basis for each group.

Table S4a – Blocks/Case (b/c) by L86x Group 1

Year	b/c G1 L861/2/3	b/c G1 L864	b/c G1 L865	b/c G1 L866	b/c G1 L86x==0
2011	1.4	2.9	14.1	27.4	0.2
2012	1.4	2.9	14.2	27.1	0.3
2013	1.7	2.8	16.8	30.5	0.1
2014	1.6	2.8	18.1	33.0	0.0
2015	1.7	2.7	19.9	31.7	0.0
2016	1.8	2.8	21.0	34.2	0.0
2017	2.3	2.8	18.9	31.9	0.1
2018	2.0	2.7	18.7	31.9	0.0
2019	1.6	2.8	18.6	31.1	0.0

Table S4b – Blocks/Case (b/c) by L86x Group 2

Year	b/c G2 L861/2/3	b/c G2 L864	b/c G2 L865	b/c G2 L866	b/c G2 L86x==0
2011	2.0	2.5	19.2	34.9	0.1
2012	2.3	2.5	20.2	38.3	0.1
2013	2.3	2.6	21.8	41.9	0.0
2014	2.4	2.7	23.7	44.5	0.0
2015	2.6	2.6	23.4	45.6	0.0
2016	2.5	2.5	20.7	43.0	0.0
2017	2.2	2.5	20.1	42.0	0.1
2018	2.4	2.5	21.5	42.7	0.1
2019	2.2	2.4	18.3	40.9	0.1

Table S4c – Blocks/Case (b/c) by L86x Group 3

Year	b/c G3 L861/2/3	b/c G3 L864	b/c G3 L865	b/c G3 L866	b/c G3 L86x==0
2011	2.0	3.1	10.5	27.5	0.5
2012	2.2	3.2	11.2	32.7	0.4
2013	2.4	3.2	11.2	25.0	0.5
2014	2.3	3.3	11.1	25.2	0.3
2015	2.4	3.5	11.5	26.5	0.2
2016	2.6	3.5	12.5	29.3	0.2
2017	2.5	3.4	11.7	29.5	0.1
2018	2.5	3.6	11.4	29.9	0.2
2019	2.4	3.6	12.4	24.2	0.2

Cases were classified by the L86x codes (L861, L862, L863, L864, L865, L866), as per the surgical pathology billing codes in Ontario (March 2020). (Schedule of Benefits, Ontario Ministry of Health and Ministry of Long Term Care, Canada, 2020) These codes are assigned on the basis of the containers in the case. To arrive at a singular code, the highest L86x code was used. For example, if the case had the codes L866 and L864, it would be classified as L866. If the case had the codes L864 and L863, it would be classified as L864. The three lowest categories (L861, L862, L863) were lumped together in the analysis.

Marked variation in the groups was seen. The largest cases (L866) varied from ~24 blocks/case (group 3) to ~41 blocks/case (group 2).

Variation of Ontario Schedule of Benefits fees by L4E workload unit

The compensation per L4E (in 2019) was higher in biopsies ($48.02/L4E unit for L864 cases) than large resections ($27.14/L4E unit for L866 cases). The Ontario Schedule of Benefits fees range suggests take home pay differs dramatically for the same amount of work, as per L4E units.
