# Supplementary material for: Pathologist workload, work distribution and significant absences or departures at a regional hospital laboratory
Source: PLoS One. 2022 Mar 25;17(3):e0265905. doi: 10.1371/journal.pone.0265905 (PMC8956155; doi:10.1371/journal.pone.0265905)
Supplement: S1 Appendix — (DOCX) [file pone.0265905.s003.docx]

# Appendix A

The FTE adjustments were calculated as follows:

1) tabulating the L4Es done by all members of each group to obtain “group work” (e.g. “L4E_group1” is the total L4Es done by all members of group 1)

2) obtaining the “work fractions”, i.e. the fraction of all work done by each group, e.g. f_group1=L4E_group1/(L4E_group1+L4E_group2+L4E_group3)

3) finding the the “ideal number of FTEs per group”; multiply the “work fraction” by the total number of FTEs in group 1, 2 and 3

4) subtracting the number of FTEs from the ideal number of FTEs to obtain the FTE adjustment for equal work (“Robin Hood” FTEs)
